# Indirect, but not direct, lateral habenula projections to ventral tegmental area regulate the long-term consequences of neuropathic pain

**DOI:** 10.1038/s41386-025-02111-5

**Published:** 2025-04-22

**Authors:** Emily D. Prévost, David H. Root

**Affiliations:** Department of Psychology and Neuroscience, University of Colorado Boulder, Boulder, CO USA

**Keywords:** Neuroscience, Chronic pain

Chronic pain, in which pain persists or recurs for more than 3 months, is a major cause of suffering and disability worldwide. Chronic pain caused by an insult to the somatosensory nervous system is known as neuropathic pain. Individuals affected by prolonged neuropathic pain often experience cognitive and emotional disturbances such as pain catastrophizing, anxiety, depressive symptoms, and fear or avoidance of movement and injury [1].

Researchers have found that pain-induced cognitive and affective changes correspond to functional changes in brain regions involved in pain processing, attention to pain, emotion, motor activity, and top-down pain inhibition [1]. Among these regions, the lateral habenula (LHb) has recently been of interest for its roles at the intersection of pain processing and pain-related cognitive and affective functions.

**Fig. 1:**
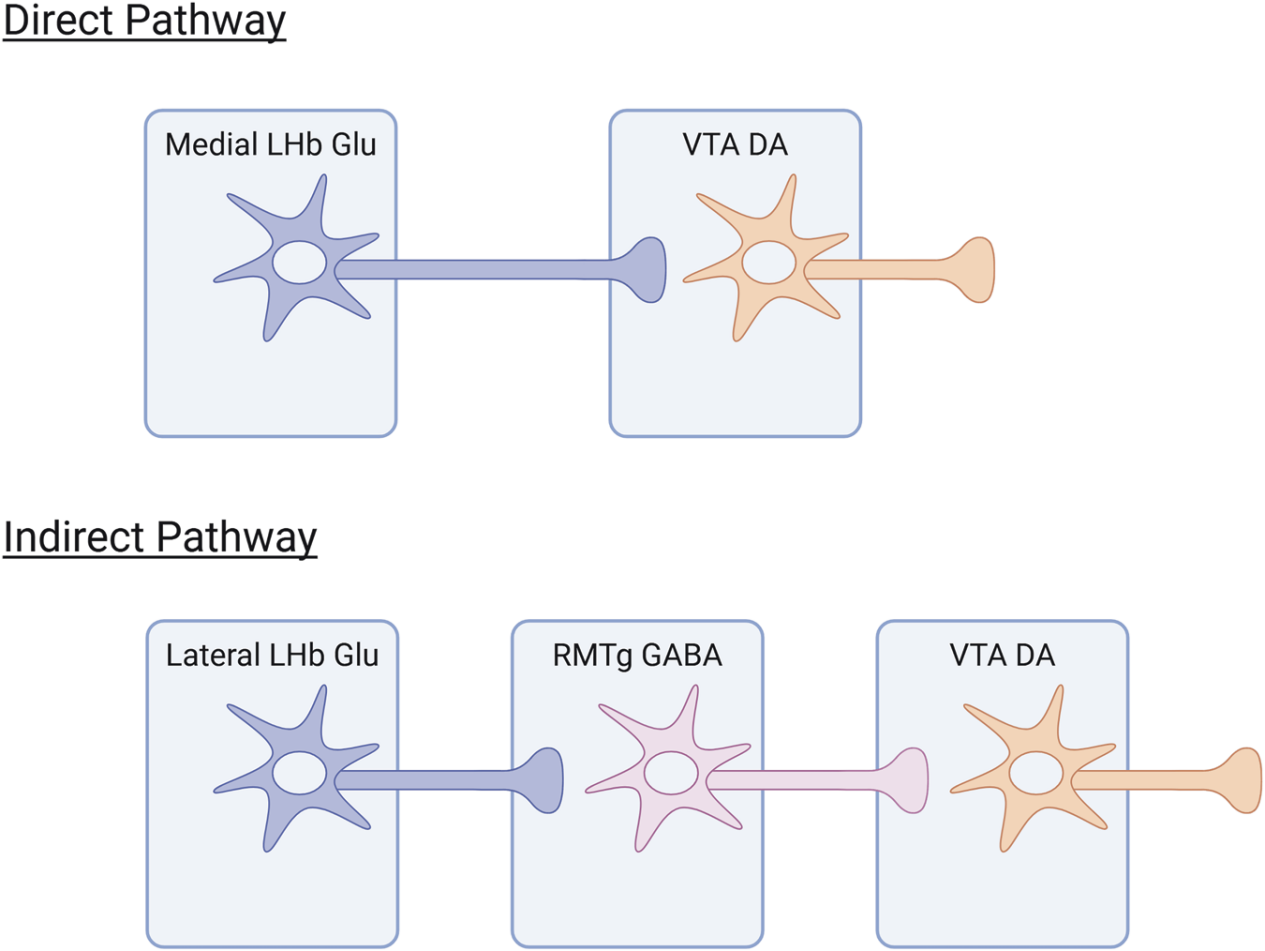
Proposed circuits for direct and indirect LHb modulation of VTA dopamine signaling. The direct pathway is hypothesized to exert excitatory control while the indirect pathway is hypothesized to exert inhibitory control. Adapted from Liu et al. [2].

LHb is a crucial processor of motivational and cognitive information, and one of its major targets is the ventral tegmental area (VTA). VTA is involved in learning, motivation, and importantly, the development of depressive symptons and cognitive dysfunction. LHb controls dopamine release from VTA via direct and indirect pathways. In the direct pathway, glutamatergic LHb neurons form monosynaptic connections with dopaminergic, GABAergic, and other VTA cell-types [2]. Activation of this pathway is aversive and dependent on dopamine receptors in the prefrontal cortex [3]. In the indirect pathway, LHb^Glu^ neurons synapse onto rostromedial tegmental nucleus (RMTg) neurons which in turn provide feedforward GABA release onto VTA^DA^ neurons (Fig. 1). While activation of this pathway is also aversive [4], the direct and indirect pathways exert differential control on the dopaminergic system.

In the present study, Liu et al. find that the indirect LHb^Glu^ → RMTg^GABA^ → VTA^DA^ pathway is a converging circuit for chronic pain processing, depressive symptoms, and cognitive impairments in neuropathic pain. Furthermore, the authors identify anatomical and electrophysiological differences between the indirect and direct pathways which may underlie their different functions [2].

Liu et al. used a model of neuropathic pain resulting in distinct short-term and long-term symptoms. Mice received spared nerve injury surgery in which the sciatic nerve was partially lesioned and were evaluated at two- and six-weeks post-surgery for mechanical and thermal pain sensitivity, depressive-like symptoms in the forced swim and tail suspension tests, and cognitive impairments in short- and long-term memory assays. While mice displayed signs of heightened pain sensitivity at both two and six weeks compared to sham controls, depressive symptoms and cognitive impairments were only observed at six weeks. A c-Fos immunoassay revealed LHb and other regions were activated in response to long-term neuropathic pain but the VTA lacked c-Fos activity. Based on these and prior results, the authors first examined the role of LHb^Glu^ neurons at the convergence of neuropathic pain processing and impairments in cognition and affect. Using in vivo calcium imaging, Liu et al. found that long-term neuropathic pain decreased LHb activity during struggle bouts in the tail suspension test and increased LHb activity during mechanical pain assays. Chemogenetic inhibition of LHb neurons decreased pain sensitivity, depressive symptoms, and cognitive impairments. From this, the authors concluded that long-term neuropathic pain enhances the excitability of LHb neurons that results in nociceptive, affective, and cognitive consequences of long-term neuropathic pain.

The authors then examined the direct and indirect LHb → VTA pathways. Neither chemogenetic nor optogenetic activation of the direct LHb → VTA pathway influenced pain sensitivity, depressive symptoms, or cognition in neuropathic pain mice. However, chemogenetic and optogenetic inhibition of the indirect LHb → RMTg → VTA pathway decreased pain sensitivity, cognitive deficits, and depressive symptoms in long-term neuropathic pain mice. Conversely, chemogenetic activation of the indirect pathway increased pain sensitivity, cognitive deficits, and depressive symptoms in sham mice.

Given the different effects of direct and indirect pathways on the consequences of neuropathic pain, the authors then anatomically compared the two pathways. First, indirect RMTg projections largely innervated DAergic VTA neurons and were more numerous than direct LHb projections, while direct LHb projections equally innervated DAergic and GABAergic neurons. Second, among LHb neurons, indirect RMTg projections increased c-Fos expression in response to long-term neuropathic pain while direct VTA projections did not. Finally, the proportion of burst-firing LHb neurons increased by factor of about 2.5 in indirect projections while the proportion of direct projections did not change. These results provide strong support for a role of the indirect LHb → RMTg → VTA pathway in the enduring consequences of neuropathic pain.

Together, Liu et al. employed an effective combination of behavioral, chemogenetic, optogenetic, and electrophysiological techniques to dissect the direct and indirect pathways from LHb to VTA and their roles in pain-induced affective and cognitive impairments. Their results provide compelling evidence that RMTg GABAergic neurons serve as an essential inhibitory relay between LHb glutamatergic neurons and VTA dopaminergic neurons in chronic pain processing and the emotional and cognitive consequences thereof, and that a proportional increase in burst-firing neurons in the disynaptic circuit likely underlies these detriments. Given that LHb burst firing has been strongly linked with depression [5], their findings provide new insights into LHb and potentially point to burst-firing patterns as potential targets for neuropathic pain therapies. While the direct LHb → VTA pathway mediates aversion [3], its function is dissociable from those involved in the enduring cognitive and emotional consequences of neuropathic pain.
